# Unmasking the Silent Threat: Meleney's Synergistic Gangrene in a Healthy Young Woman Without Predisposing Factors

**DOI:** 10.7759/cureus.63849

**Published:** 2024-07-04

**Authors:** Prabhat Nichkaode, Shriya Haval

**Affiliations:** 1 Surgery, Dr. D. Y. Patil Vidyapeeth, Pune, IND; 2 General Surgery, Dr. D. Y. Patil Vidyapeeth, Pune, IND

**Keywords:** polymicrobial organisms, antibiotics, debridement, necrotizing fasciitis, meleney’s gangrene

## Abstract

A rare rapidly-spreading necrotizing infection of the skin and soft tissues, Meleney's synergistic gangrene is characterized by a synergistic infection with both staphylococci and microaerophilic streptococci. This report presents a case of Meleney's synergistic gangrene in a young female patient with no comorbidities and no surgical history who was initially misdiagnosed as a case of perineal abscess and later after the culture report and course of the spread of infection, it was diagnosed as a case of Meleney's synergistic gangrene. The patient underwent serial debridements with a combination of broad-spectrum antibiotic cover followed by secondary closure of the wound and the patient was followed up after three months post-discharge and showed full recovery with no recurrence of infection.

## Introduction

Meleney's gangrene is a deadly infection that spreads rapidly and causes necrosis of the abdomen's skin and subcutaneous tissue, sometimes even reaching the underlying muscle. It has been identified and treated as a potentially fatal uncommon disorder since the 18th century [1] and has gone by numerous names, including hospital gangrene, necrotizing fasciitis, and Fournier's gangrene [2]. Its microbiological effectors were initially identified by Drs. Meleney and Brewer in 1926, and Meleney further categorized them in 1931 [3]. This is a quite uncommon necrotizing infection of the anterior abdominal wall that usually manifests itself two weeks following mild trauma or surgery [4]. A combination of proper antibiotic coverage and vigorous debridement is the main treatment for Meleney's gangrene. It can result in fatal consequences and other serious problems if left untreated. This case report illustrates an unprecedented instance of Meleney's gangrene in a previously healthy female, highlighting the atypical presentation and the successful management strategy employed. This case report aims to contribute to the existing knowledge of Meleney’s gangrene by offering a comprehensive understanding of the early recognition and multidisciplinary approach that are necessary for achieving the best possible patient outcomes.

## Case presentation

**Figure 1:**
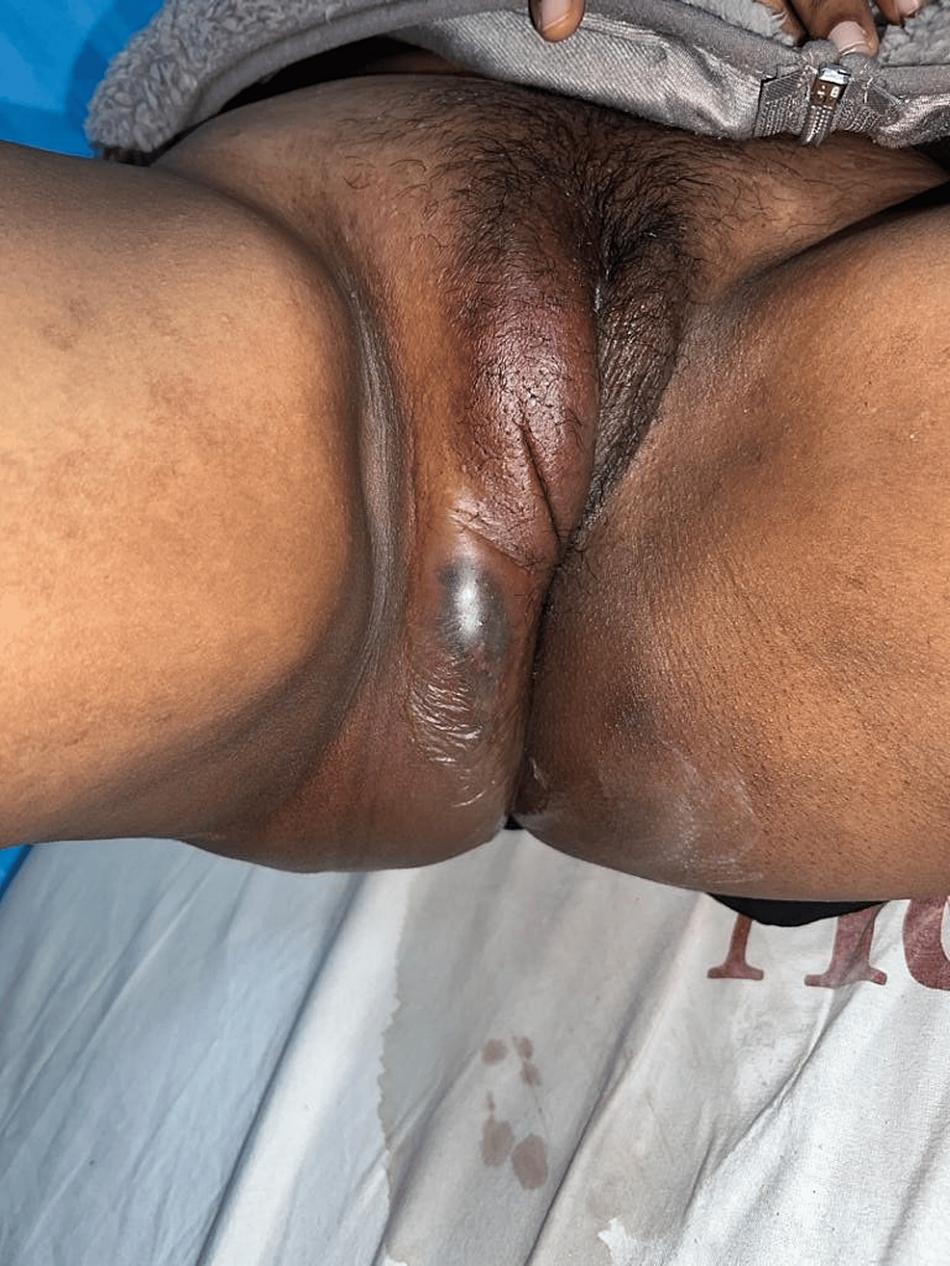
Clinical photo of the patient on presentation

A 30-year-old woman with no history of prior surgery, co-morbidities, or steroid use arrived as a casualty with severe pain and swelling in the perineal region and a foul-smelling discharge. On physical examination and initial workup, vital signs were oxygen saturation on room air was 94%, blood pressure was 90/54 mmHg, heart rate was 118 beats per minute, respiratory rate was 20 breaths per minute, and temperature was 98.5°F. Local examination revealed a tender and fluctuant swelling of size 15x10 cm was present in the right perineal region, extending up to 2 cm away from the vulva. Local rise of temperature was present. A 4 x 3 cm area of skin excoriation is present over the right gluteal region. Thick, yellowish, purulent, foul-smelling pus discharges from swelling (Figure 1). Greenish patches were seen over the right thigh upper aspect and right labia majora without any tenderness or local rise in temperature and no crepitus. The per rectal examination is normal, and the per vaginal examination is normal.

Laboratory tests revealed increased C-reactive protein (CRP) (308 mg/dL) and white blood cell count (16,080/mm^3^).

Computed tomography (CT) of the perianal region revealed an approximately 3.4 x 3.7 × 5.1 cm (volume: 40-50 cm)-sized abscess or located collection noted in the perianal region on the right side. Fat stranding with air foci in the right-sided ischiorectal fossa. Possibility of right-sided ruptured perianal abscess with its extension.

Given the severity of the presentation and imaging findings, a diagnosis of necrotizing fasciitis secondary to perineal abscess was made, and the patient was taken for emergency surgical exploration.

**Figure 2:**
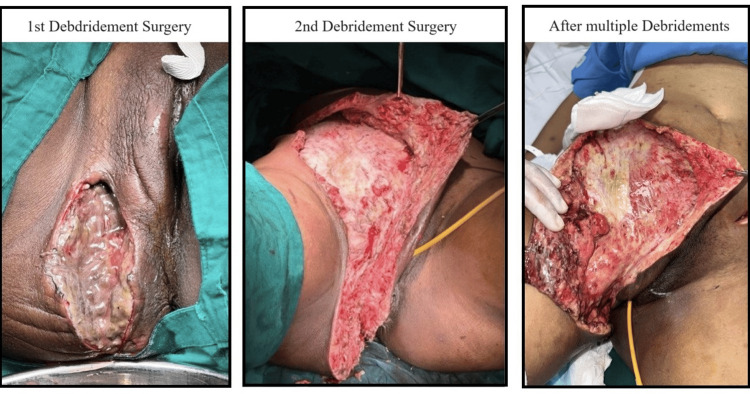
Clinical image of the wound after serial debridements and dressings

Surgical findings revealed intraoperatively a 150-cc-thick pus with extensive necrosis of the subcutaneous tissue and fascia up to the ischiorectal fossa (Figure 2). During surgery, it was found that the extent of the abscess was much greater than the CT findings.

By postoperative day 2, the infection had extended upwards toward the right inguinal region and right lower abdomen, thus the patient was taken up for extensive re-debridement surgery (Figure 2).

Cultures from the affected tissue grew *Staphylococcus aureus* and *Streptococcus pyogenes*, consistent with a synergistic infection.

The patient underwent multiple surgical debridements to remove necrotic tissue and pus (Figure 2). She was started on broad-spectrum antibiotics, including piperacillin, tazobactam, and clindamycin, and thorough dressings were done in the ward. Given the severity of the infection, she was later switched to tigecycline intravenously, based on culture sensitivity results.

Outcome and follow-up

**Figure 3:**
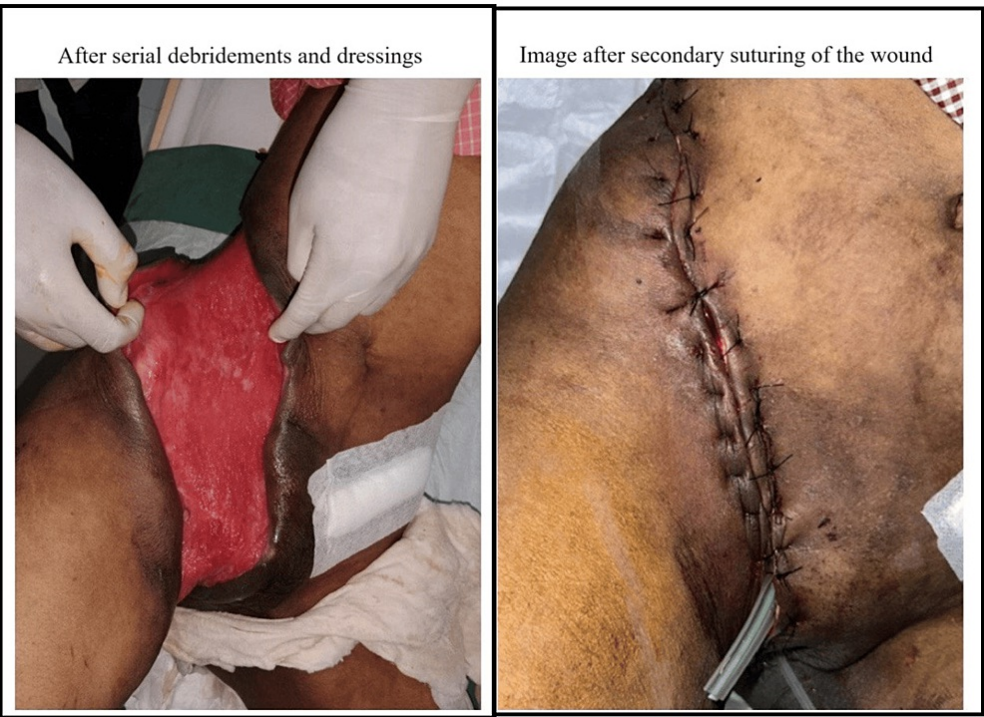
Clinical image of the wound getting better after the final surgery

Over the next few weeks, the patient showed gradual improvement (Figure 3). The wound was managed with daily dressings. She completed a six-week course of antibiotics. Immunofluorescence (IF) was used with the help of the microbiology department to help visualize live organisms from the IF machine, which revealed no live organisms. Together with a negative culture report, all antibiotics were stopped and the patient was posted for secondary suturing of the wound (Figure 3). The patient was subsequently discharged 10 days after secondary suturing and was followed up for regular dressings in the ward.

**Figure 4:**
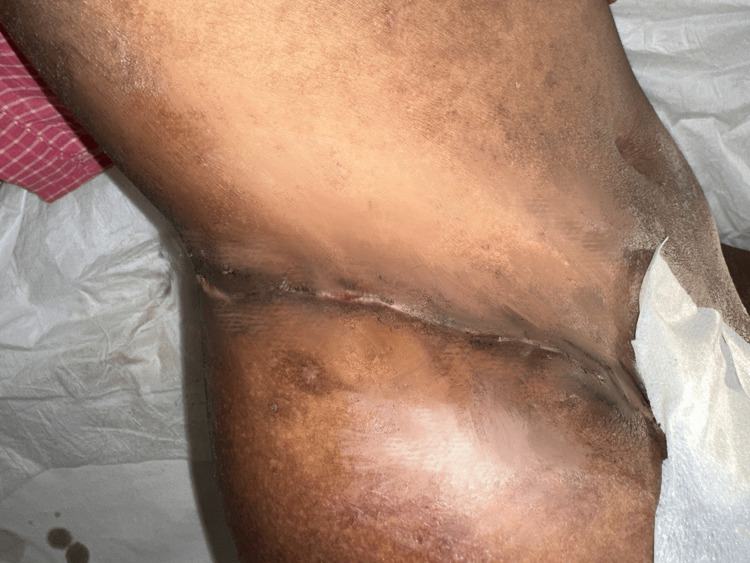
Clinical image after one month of discharge from hospital

At the one-month (Figure 4) and three-month follow-up, the patient had fully recovered, with a completely healed wound and no signs of recurrent infection.

## Discussion

The general term for Meleney's gangrene is "necrotizing fasciitis." Necrotizing fasciitis is a potentially fatal illness with a 34% fatality rate [1]. Widespread inflammation of the skin, deep fascia, and soft tissues are the hallmarks of necrotizing fasciitis. Additionally, toxemia caused by a mix of gram-negative, coliform, and anaerobe bacteria is present [5]. Major risk factors include diabetes, drug addiction, alcoholism, obesity, malnutrition, cancer, immunodeficiency, and other long-term medical conditions [6]. Meleney's gangrene is difficult to diagnose in the early stages because of its appearance, which might be mistaken for cellulitis or an abscess [7]. It is comparable to gas gangrene, when comparing Meleney's gangrene to gas gangrene, conventional X-ray imaging shows no crepitus or gas. Early detection and treatment of Meleney's gangrene during the first 24 hours can lower mortality from 70% to 35%, according to Freischlag et al. [8]. It is currently suggested that all necrotizing infections be referred to as necrotizing soft tissue infections (NSTIs) and that treatment planning and diagnosis be done using the same methods. This will encourage quicker diagnosis and treatment, both of which are necessary to enhance outcomes and reduce death rates in NSTI-affected individuals [9].

There is an urgent surgical need. Prognosis improvement depends on early diagnosis, which is usually achieved by clinical procedures. The first line of treatment for necrotizing fasciitis is aggressive surgical debridement, which is followed by targeted antibiotic therapy [10]. Meleney's gangrene may not usually present in an immunocompromised or diabetic state. Chhetry et al. [11] reported a case in which Meleney's gangrene occurred in an immune-competent individual infected with the monomicrobial organism. Makhdoomi et al. [12] reported that an immunocompetent patient suffering from Meleney's gangrene recovered promptly and with decreased morbidity. Meleney's gangrene is caused by polymicrobial agents, specifically microaerophilic nonhemolytic streptococcus, beta-hemolytic Staphylococcus, anaerobes, and coliform organisms. Meleney's gangrene is caused by hemolytic Staphylococci in the majority of recorded cases, with nonhemolytic Streptococcus accounting for 20% to 28%. The remaining 14%-35% of cases are caused by polymicrobial organisms. Pseudomonas, coliform organisms, diphtheroid, proteus, and Bacteroides are some of the other documented polymicrobial organisms [13]. Polymicrobial organisms are typically introduced in subcutaneous tissue after surgery or trauma. Following the first introduction of bacteria, the infection progresses quickly through the subcutaneous fascia. Following inoculation, bacteria produce proteolytic enzymes, hemolytic enzymes, and bacterial toxins. It induces nonspecific inflammatory alterations, arteriolitis, and small-vessel thrombosis. During this time, Meleney's gangrene will have few skin symptoms. Following thrombosis of small blood vessels, there will be patch gangrene of the skin, followed by secondary gangrene of the entire abdominal wall. In an immunocompromised person, the organisms travel via subcutaneous tissue and cause small vessel thrombosis, followed by tissue necrosis [13].

## Conclusions

Most of Meleney's gangrene signs and symptoms go unnoticed at first, which increases mortality needlessly. Prompt surgical debridement with adequate antibiotics is the cornerstone treatment for Meleney’s gangrene. Delayed presentation of Meleney’s gangrene increases both morbidity and mortality. This case underscores the importance of recognizing Meleney's synergistic gangrene in the differential diagnosis of NSTIs, even in young, healthy individuals without surgical history. Early intervention and appropriate management can lead to favorable outcomes.
